# The impact of learning communities on interpersonal relationships among medical students

**DOI:** 10.3402/meo.v21.32958

**Published:** 2016-11-01

**Authors:** Eve Privman Champaloux, Meg G. Keeley

**Affiliations:** Department of Pediatrics and Office of Student Affairs, University of Virginia School of Medicine, Charlottesville, VA, USA

**Keywords:** learning communities, interpersonal relationships, social networks

## Abstract

**Background:**

Medical students at the University of Virginia (UVA) are mentored and learn within the framework of a four college learning community. Uniquely, these learning communities are used to organize the third-year clerkship rotations.

**Methods:**

Students were surveyed after their first pre-clinical year and after their clerkship year to determine what the effect of the learning community was on their social and educational interpersonal relationships.

**Results:**

Students knew a higher percentage of their college mates after completing their third-year clerkships within the framework of the college system. Students chose peers from within the college system for social and educational interpersonal scenarios statistically more often than what would be expected at random. Small group learning environments that were not formed within the framework of the college system at UVA did not have the same effect on interpersonal relationships, indicating that learning communities are uniquely able to provide a context for relationship building. Students felt more positively about the social and educational effects of the college system after the clerkship year, with a corresponding increase in the strength of their interpersonal bonds with their college peers.

**Conclusion:**

This work is the first to investigate the effects of learning communities on interpersonal relationships among medical students and finds that learning communities positively impact both social and educational medical student bonds.

Increasing numbers of medical schools are utilizing a framework of learning communities (LCs) to organize curricular, community service, advising, and social experiences for students (1). Over 100 medical schools report incorporating LCs into their educational programs (2). At the University of Virginia (UVA), medical students are assigned to one of the four colleges headed by an advisory dean. Colleges are subdivided into groups who meet regularly with mentors as part of the Clinical Performance Development (CPD) course. Other learning activities occur in small groups during the pre-clerkship curriculum, including team-based learning and anatomy dissection. These are deliberately formed to preclude overlapping membership.

A unique aspect of the UVA LC framework is that students complete the clerkship year as a college. Each LC has a prescribed sequence of clerkship blocks so that all students know their schedule from the start of medical school and students on clinical teams are members of the same college. Clerkship faculty know that all students on their rotation have had the same clerkship experience which improves orientation efficiency. In addition, a shared schedule permits the continuation of the small-group curriculum in the clerkship year. This is unlike most medical schools where the students each have their own individual sequence of clerkships and work with different subgroups of classmates on different clerkships. Students complete their clerkship training at the University of Virginia Health System academic teaching center and spend an average of 14 weeks at several satellite sites and the private offices of affiliated faculty.

Studies show that medical students’ perception of their learning environment may impact their quality of life and the presence of LCs is associated with a more positive learning environment (3, 4). However, the social impact of LCs has not been studied among medical students. We hypothesized that LCs impact the interpersonal relationships of medical students in both educational and social settings. In addition, we hypothesized that completing the third-year clerkships within the college framework would strengthen student bonds.

## Methods

First-year and third-year UVA medical students were surveyed during the 2015–2016 academic year in accordance with the institutional review board. Ninety-one students responded after their first full year of medical school and 84 students responded after their third-year clerkships. Students were provided with scenarios and then asked to identify which medical student from their class they would contact. These included social (e.g., who would you contact to go out to lunch) and educational scenarios (e.g., who would you contact to be a study buddy for an exam). Students were asked whether the person they selected for each scenario was a student they met at orientation, a student from their college, a student from their six-person CPD group (a subset of their college), a student from one of their six-person team-based learning (TBL) groups, or a student from their four-person anatomy group. Students were also asked to rate the impact of the college system on their educational and social experiences using a Likert scale and to estimate how accurately they could identify which students were in their college. Qualitative data were also collected about the social effects of the college system. Statistics were performed using Graphpad Prism 6 and SPSS 23. Graphs of data are mean ± standard error of the mean. Statistical significance is indicated as *p<0.05, **p<0.01, ***p<0.001, and ****p<0.0001.

## Results

**Fig. 1:**
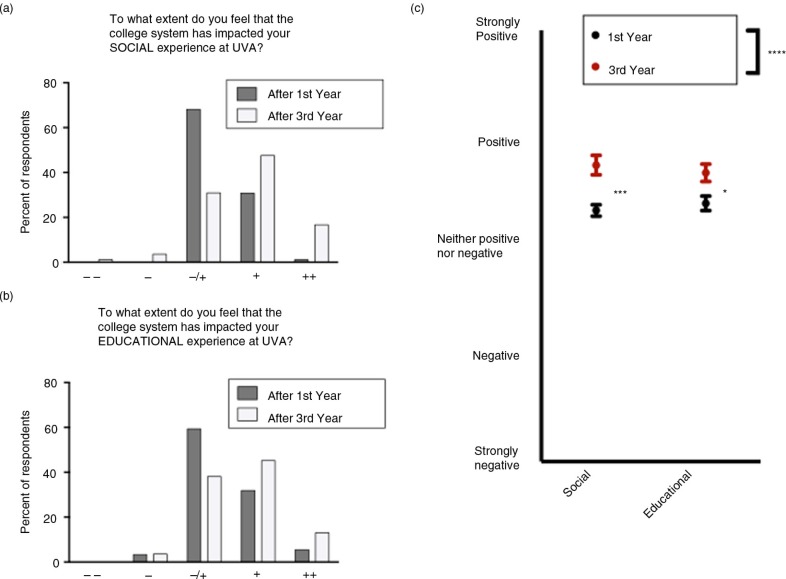
The effect of the University of Virginia college system on the social and education experiences of medical students. The majority of students felt that the college system had neither a positive nor a negative effect after first year, and that it had a positive effect after third year, on both their (a) social and (b) educational experience. (c) Students felt more positive about both the educational and social impacts of the college system after third year.

Students were asked to rate the effect of the college system on their social and educational experiences using a Likert scale (Fig. 1). After first year, the majority of students felt neutral about the effects of the college system on both social and educational aspects of their experience. After third-year clerkships with their college classmates, students felt more positive about both the social and educational experiences (Mann-Whitney *t*-tests, *p<*0.01).

**Fig. 2:**
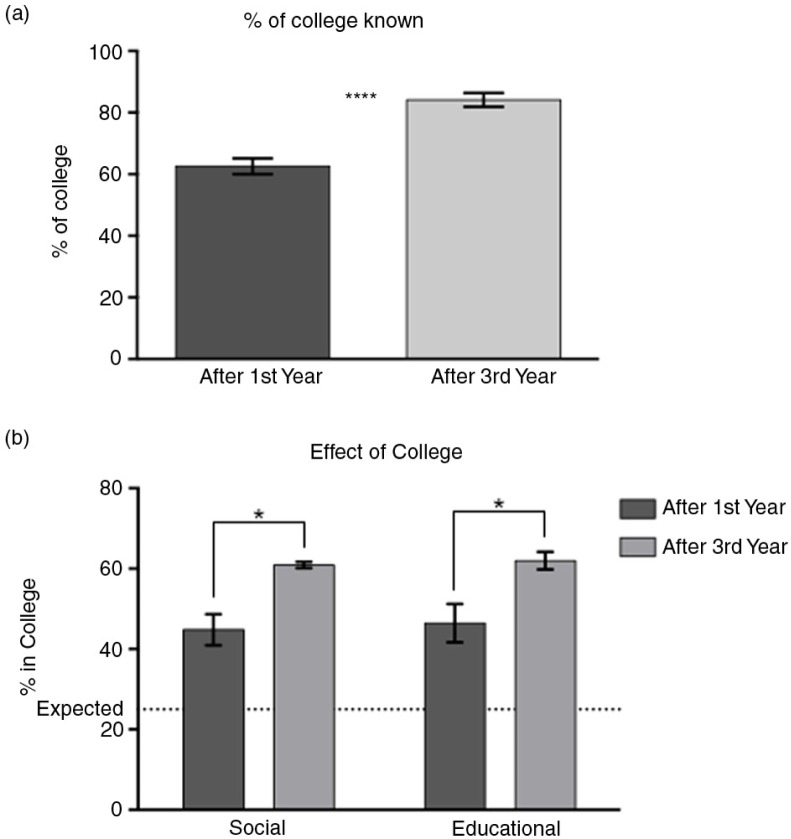
Students have more interpersonal relationships with students in their college and this effect increases after third year. (a) Students can identify a higher percentage of their college mates after third year. (b) Students would call upon students in their college more than expected (dotted line) for both social and educational scenarios, and this effect increases after third-year clerkships.

Students knew a higher percentage of members of their college after third-year clinical rotations than after first year (Fig. 2a, *t*-test, *p<*0.0001). When asked which students they would call upon for various social and educational scenarios, students called upon members of their college significantly more than would be expected (25%) at random (Fig. 2b, *p<*0.05 for all columns). This effect intensified after the third year of medical school (two-way analysis of variance (ANOVA), significant main effect of year, *p=*0.0013).

**Fig. 3:**
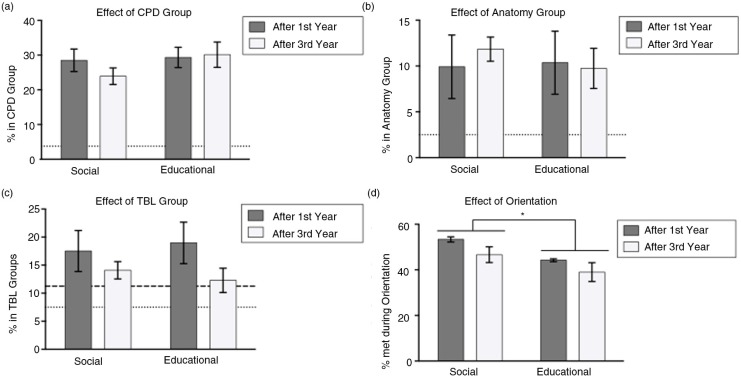
Students have more social and educational interactions with students from their college-derived small groups than their randomly assigned small groups. (a) Students rely on their CPD group more than expected (dotted line) for both social and educational scenarios equally after first and third years. (b) Students do not rely on their anatomy group more than expected (dotted line). (c) Students do not rely on their TBL group more than expected after either first year (dotted line) or third year (dashed line). (d) Students rely on people they met during orientation more for social than educational scenarios.

Students called upon members of their CPD group (a subset of their college) more frequently than expected (3.75%) for both scenario types (Fig. 3a, *p<*0.05). However, the anatomy and TBL small groups that were formed from the entire class and deliberately not overlapping in membership with the CPD groups or each other (Fig. 3b and c) did not show higher than anticipated rates of social or educational bonds. Students form more social than educational bonds with students they meet at orientation (Fig. 3d, two-way ANOVA, main effect of scenario type, *p=*0.0167).

## Discussion and conclusions

Medical students make lasting friendships early in medical school. Despite the stressors associated with medical education, there is evidence that the social well-being of students can increase during the first year as they engage in similar activities and their social world condenses (5). The LC framework capitalizes on this sense of membership, influence, and shared emotional connections through intentionally formed active learning groups (6). Positive early relationships can exert powerful influences on professional identity formation as students progress through their medical education (7). This study demonstrates a strong impact of the college system on both the social and educational interpersonal relationships of UVA medical students, which is not seen in other small group learning outside the LC framework. The unique college-based third-year rotation system extends this sense of community and emphasizes team-oriented practice, encourages mutual support through often challenging situations, and promotes social integration and resilience (8).

When asked their opinion on the effect of the college system on their social and educational experience, some of the comments indicated that the social benefits were especially helpful for introverts. After first year, the students indicated that the system could be improved with more structured college social events, indicating a desire to use the college system as a framework for friendship building. After third year, the comments were overwhelmingly positive with very little room for improvement. Students indicated a strong sense of companionship that benefited both their medical education and social lives. ‘Being with the same group of people throughout the third-year clerkships provided a sense of camaraderie and group of people with similar experiences to talk with about any problems with the rotations’. Students, especially introverts, were ‘able to focus on developing closer relationships with a smaller group of individuals’. Students also appreciated the greater availability of their advising deans and the opportunities for longitudinal relationships with their mentors.

The LC framework at UVA promotes the development and maintenance of social and educational interpersonal relationships among medical students. The unique use of this system during the third-year clinical clerkships addresses process management issues of scheduling and monitoring performance development, and also encourages social networks that support student education and well-being. Introverted students report a particular benefit. Future research involving students at other institutions with different LC frameworks will help further elucidate these effects.
